# G-spots cause incorrect expression measurement in Affymetrix microarrays

**DOI:** 10.1186/1471-2164-9-613

**Published:** 2008-12-18

**Authors:** Graham JG Upton, William B Langdon, Andrew P Harrison

**Affiliations:** 1 Department of Mathematical Sciences, University of Essex, Wivenhoe Park, Colchester, Essex, CO4 3SQ, UK; 2 Departments of Mathematical and Biological Sciences, University of Essex, Wivenhoe Park, Colchester, Essex, CO4 3SQ, UK

## Abstract

**Background:**

High Density Oligonucleotide arrays (HDONAs), such as the Affymetrix HG-U133A GeneChip, use sets of probes chosen to match specified genes, with the expectation that if a particular gene is highly expressed then all the probes in that gene's probe set will provide a consistent message signifying the gene's presence. However, probes that contain a G-spot (a sequence of four or more guanines) behave abnormally and it has been suggested that these probes are responding to some biochemical effect such as the formation of G-quadruplexes.

**Results:**

We have tested this expectation by examining the correlation coefficients between pairs of probes using the data on thousands of arrays that are available in the NCBI Gene Expression Omnibus (GEO) repository. We confirm the finding that G-spot probes are poorly correlated with others in their probesets and reveal that, by contrast, they are highly correlated with one another. We demonstrate that the correlation is most marked when the G-spot is at the 5' end of the probe.

**Conclusion:**

Since these G-spot probes generally show little correlation with the other members of their probesets they are not fit for purpose and their values should be excluded when calculating gene expression values. This has serious implications, since more than 40% of the probesets in the HG-U133A GeneChip contain at least one such probe. Future array designs should avoid these untrustworthy probes.

## Background

Microarrays are commonly used to measure gene expression. One of the most popular microarray platforms is the Affymetrix GeneChip. In GeneChip arrays probe sequences with a nominal length of 25 bases are created by photolithography. The probes are arranged in pairs: a so-called Perfect Match (PM) probe and a mismatch (MM) probe that is identical to the PM probe with the exception that the 13th base is the complement of that in the PM probe. Each pair of probes belongs to a probe set (typically of 11 or 16 probe pairs) with each probe set being intended to provide information concerning the expression of a single gene. For some genes there may be more than one dedicated probe set.

There are a number of alternative software tools for calculating a single measure of gene expression for a probe set: e.g. MAS5[1], dChip[2], RMA[3] and GCRMA[4]. To calculate the value of the expression measure, all the probes (or at least all the PM probes) in a probe set are used. However, if there are probes that are known to be liable to provide misleading information, then these should be excluded from the analysis so as to give more accurate estimates of gene expression. The existence of large datasets such as that contained in the NCBI Gene Expression Omnibus (GEO) repository [5] provides an opportunity to identify such probes. We report an analysis that unambiguously identifies a large class of more than 10 000 probes whose behaviour is not that intended. More than 40% of probesets contain one or more members of this family.

### Our approach

We have focused on the GeneChip oligonucleotide microarrays manufactured by Affymetrix. Since a major application of microarrays has been for the study of human diseases, we have concentrated our effort on data from the most popular human GeneChips, the HG-U133A arrays, though *the results apply to all GeneChip arrays*.

Within a probe set, subsets of probes may be measuring different exons and thus, potentially, different transcripts, implying that biological signals such as alternative splicing would need to be taken into account[6]. We have therefore focused on groups of probes which map uniquely to the same exon. We have downloaded from GEO more than 6000 data sets relating to about 300 Gene expression Series (GSEs) and have then calculated the standard Pearson's (product-moment) correlation coefficients between relevant probe pairs.

**Figure 1:**
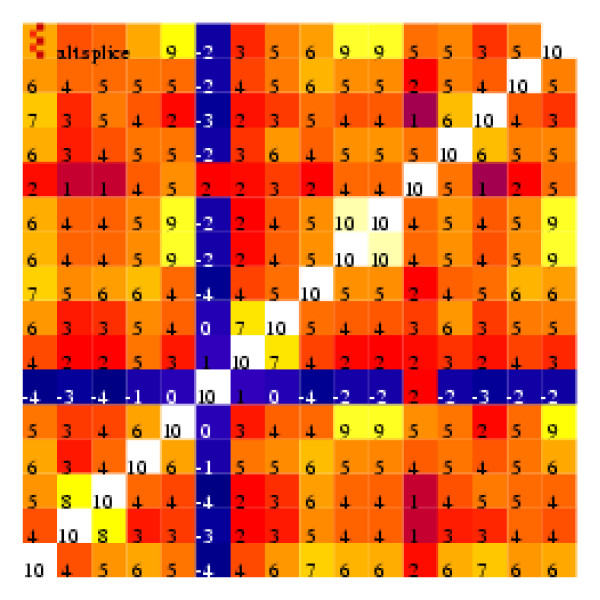
**Heat map illustrating correlation coefficients between every pair of the 16 perfect match probes that form the 31846_at probe set**.

As a visual display of the correlation coefficients between pairs of probes, with the probes selected corresponding to a single exon, we formed "heatmaps" such as that illustrated in Figure 1. In this diagram the shade in each cell represents the value of the correlation coefficient between the values shown by a particular pair of probes. The actual correlation coefficients (multiplied by 10 and rounded) are also shown. The values on the main diagonal are all 10, since the correlation coefficient for a value matched with itself is 1.

Figure 1 shows the heatmap for the 16 perfect match probes in the probeset 31846_at all of which relate to the same exon. Evidently probe 6 is not behaving in the same way as the other probes since its values have near-zero correlation coefficients when matched with the values of these other probes. Looking at other heatmaps we found that such 'misbehaving' probes were not unusual. By listing the base sequences of these unusual probes we observed that a frequent feature was a sequence of four or more guanines. That such probes are typically poorly correlated with other members of their probe set had already been noted [7] who suggested that this might be due to the formation of G-quadruplexes [8]. We will show that, although these probes are ill correlated with others in their probeset, they are well correlated with each other. This suggests that their behaviour, varying from array to array, must be a consequence of the method of preparation of the array.

We suggest that probes with sequences of four or more guanines should be ignored when analyzing the results of current GeneChip designs. We also suggest that future GeneChip designs should avoid including probes containing such sequences.

## Results and Discussion

### Results

#### Correlation coefficients between pairs of probes

**Figure 2:**
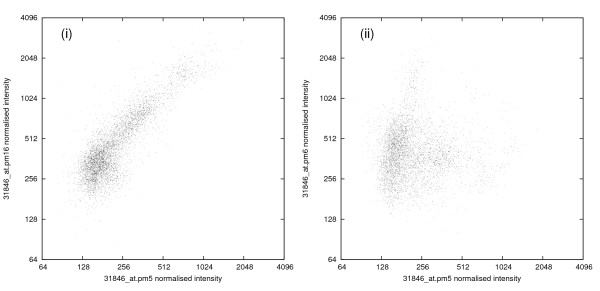
**Scatter diagrams of normalised probe intensities for two pairs of probes from probe set 31846_at (which matches the gene RHOD). **(i) Probes PM 5 and PM 16 (*r* = 0.86); (ii) Probes PM 5 and PM 6 (*r* = -0.01).

Our results use data from 6685 HG-U133A CEL files downloaded from the NCBI Gene Expression Omnibus (GEO) repository[5]. (After purified mRNA is processed and hybridised to an array, the Affymetrix scanner stores the average fluorescence intensity of each probe in the array in a data file, known as a CEL file.) The HG-U133A array contains about 22 300 probe sets matching to about 16 000 genes. After normalising each CEL file, we examined the values of the correlation coefficients between pairs of probes from within the same probe set searching for anomalies. An example is provided by the probe set 31846_at which is one of two probe sets designed to match the gene RHOD. This probe set contains 16 PM probes all drawn from the same exon and gives rise to the correlation 'heatmap' of Fig. 1. The value of the correlation coefficient between almost any pair of these PM probes is strongly positive, with the sole exceptions being that probe pm6 (the sixth of the PM probes in this probe set) has near-zero values for its correlation coefficients with all the other probes. The values giving rise to some of these correlation coefficients are indicated in the scatter diagrams in Fig. 2. Although probes 5 and 16 are separated by 192 bases their log(intensities) are highly correlated (*r *= 0.86), whereas probes pm5 and pm6, though separated by just 29 bases, have log(intensities) displaying a near-zero correlation coefficient. Near-zero correlation coefficients could occur with probes having intensities so low that they are dominated by the background 'noise' of the chip, but that is not the case in this instance since the average normalised intensities for probes pm5, pm6 and pm16 are 225, 389 and 504, respectively.

**Figure 3:**
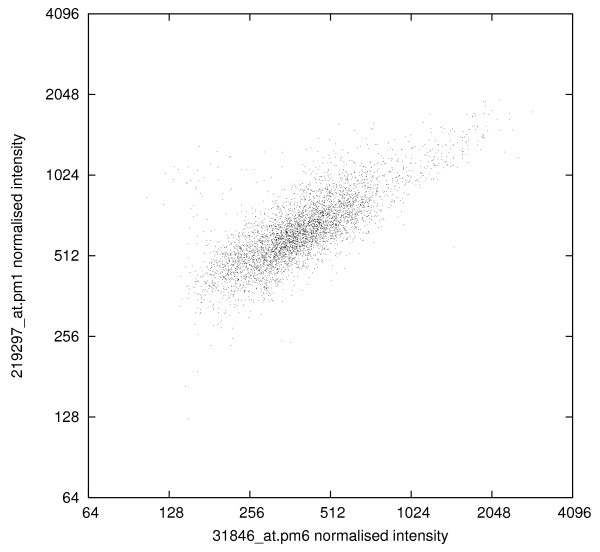
**Scatter diagram comparing probe pm6 from probe set 31846_at with probe pm1 from probe set 219297_at (*r *= 0.78)**.

To test the hypothesis that probe pm6 is related to other probes, we determined the value of its correlation coefficient with every other probe (PM and MM) in the entire array. There are 10 409 other probes (drawn from 5341 probe sets) that have correlation coefficients with probe 6 that exceed 0.8 (and no fewer than 151 that have correlation coefficients exceeding 0.95). About half these high-correlating probes are mismatch probes. As an example Fig. 3 shows the correspondence of the variation in the values of the pm6 probe with that displayed by the first PM probe in the unrelated probe set 219297_at (which was designed to measure activity of the WDR44 gene).

#### Correlation coefficients between the values of probes containing sequences of guanines

Upon listing the probes most highly correlated with probe pm6 from the 31846_at probe set (base sequence TCCTGGACTGAGAAAGGGGGTTCCT) it becomes apparent that there is a common theme: each probe contains a sequence of four or more consecutive Gs. For example, the pm1 probe in 219297_at (used in Fig. 3) begins with six Gs (GGGGGGATAGTCTTGTTTCTAGCTT). By contrast, in 31846_at, probes pm5 (GAACTCCACTGCAACAGACGGGCGC) and pm16 (TTCCCACCTGTCATACTGGTAACTG) contain sequences of only 3Gs and 2Gs, respectively.

Given that the high values of the correlation coefficients are a consequence of sequences of guanines in the probe sequence, two questions that immediately arise are 'Is the *location *within the probe of the consecutive run of guanines relevant?' and 'Is the *number *of consecutive guanines relevant?'. To get clear answers to these questions we focus on probes that have only one sequence of two or more guanines. We will refer to the location of the sequence within the probe as the *G-spot *and we now examine how the values of the inter-probe correlation coefficients are affected by the location and length of the G-spot.

#### The effect of the location of the G-spot

We will use probes containing a single sequence of exactly four guanines to demonstrate that the location of the G-spot within the probe has a considerable bearing on the value of the correlation coefficient. Let *l *denote the first base of the G-spot (so that, for these probes, *l *= 1, 2,...,22). For each value of *l*, Table 1 reports the number of probes of this type and the average value of the correlation coefficient for pairs of probes both of this type.

**Table 1:** In 4G-probes, the effect of the location of the G-spot on the average value of the correlation coefficient.

Location of G-spot, *l*	1	2	3	4	5	6	7	8	9	10	11
Number of probes	331	173	220	229	265	203	225	224	218	340	187
Average correlation	0.68	0.38	0.36	0.38	0.30	0.34	0.40	0.41	0.47	0.59	0.49
Location of G-spot, *l*	12	13	14	15	16	17	18	19	20	21	22
Number of probes	185	207	181	194	234	235	244	251	284	224	250
Average correlation	0.55	0.60	0.60	0.59	0.58	0.56	0.56	0.51	0.46	0.44	0.45

(*l *= 1 means that both probes have a 5' end that starts with GGGG; *l *= 22 means that both probes have a 3' end that finishes with GGGG).

Table 1 shows distinct design preferences on the part of Affymetrix since probes starting GGGG are relatively common (an unfortunate choice under the circumstances) whereas cases where the GGGG sequence straddles the central probe (i.e. probes with the GGGG sequence commencing at one of locations 10 to 13) are relatively infrequent. For all values of *l *the average value of the correlation coefficient for pairs of probes with G-spot at l is significantly greater than zero indicating the pervasive nature of these unwanted correlations. The overall maximum is at *l *= 1 corresponding to the G-spot being at the 5' end (the free end) of the probe.

**Figure 4:**
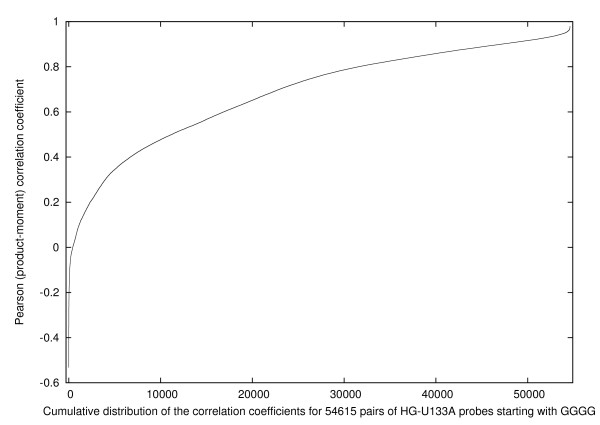
**The distribution of the correlation coefficient for pairs of probes that begin with the sequence GGGG and contain no other runs of Gs**.

Further detail is provided by Fig. 4 which shows the cumulative distribution of the 54 615 = (331 × 330/2) individual correlation coefficients for pairs of probes having *l *= 1. The figure demonstrates that fewer than 1% of these probe pairs have negative correlation coefficients, whereas 14% have correlation coefficients that exceed 0.9. Such high correlation coefficients would only be expected between the relatively few pairs of probes from related genes.

#### The effect of the length of the G-spot

We next examine how varying the number of consecutive guanine bases affects the value of the correlation coefficient. For simplicity and since the correlation coefficient is greatest when the G-spot is at the 5' end of the probe, in this section all the probes start with the G-spot. Table 2 demonstrates that, while the correlation coefficient between values of probes beginning with exactly three guanines is appreciably greater than zero, it is pairs of probes beginning with four or more guanines for which the correlation coefficient attains remarkably high values. As further confirmation we looked also at probes beginning with the sequence GGXGG (where X is any base other than G). The average value of the correlation coefficient amongst pairs of these probes was 0.06, confirming that in order to obtain high correlation coefficients *consecutive *guanines are required.

**Table 2:** Average values of the correlation coefficient between pairs of probes that each has its single sequence of *k *Gs starting with the first base

Length of starting sequence, *k*	2	3	4	5	6	7
Number of probes	5189	1279	331	67	11	5
Average correlation	0.03	0.15	0.68	0.83	0.91	0.94

#### Correlation coefficients for probes having different locations for their G-spots

Returning to probes containing a single sequence of four guanines, Table 3 displays the average values of the correlation coefficients between two such probes, when one has its G-spot at the start of the probe (*l *= 1) and the other does not. Whilst all these averages considerably exceed zero, their peak value occurs when both probes start with a G-spot (that is to say the correlation coefficient is greatest when both probes have their G-spot located at the free end of the probe).

**Table 3:** Average value of the correlation coefficient between pairs of 4G-probes where one probe has its G-spot at location 1 and the other has its G-spot at location *l*

G-spot in 2^nd ^probe, *l*	1	2	3	4	5	6	7	8	9	10	11
Average correlation	0.68	0.30	0.29	0.28	0.25	0.28	0.29	0.28	0.29	0.33	0.28
G-spot in 2^nd ^probe, *l*	12	13	14	15	16	17	18	19	20	21	22
Average correlation	0.31	0.32	0.31	0.29	0.30	0.28	0.26	0.23	0.22	0.19	0.19

**Figure 5:**
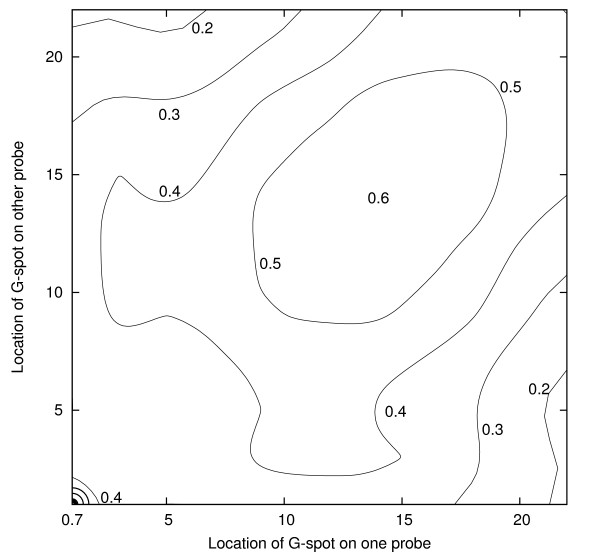
**Contour diagram showing how the average value of the correlation coefficient varies with the location of G-spot for pairs of probes (each with a single sequence of four guanines). **The values in Table 1 correspond to the main diagonal and those in Table 3 to two edges. The maximum is at the bottom left.

For probes containing a single sequence of four guanines, Tables 1 and 3 provide information concerning 43 of the 253 average correlation coefficients corresponding to pairs of values of *l*. Fig. 5 provides a contour diagram that gives an overview of the entire correlation surface. Denoting the two values of *l *by *l*_1 _and *l*_2 _there is a sharp peak at *l*_1 _= *l*_2 _= 1, a ridge along *l*_1 _= *l*_2_, with a secondary peak near *l*_1 _= *l*_2 _= 14 and a general decrease as *l*_1 _- *l*_2 _increases. This is a consistent effect and suggests that the locations of the G-spots on the probes (*l *= 1 corresponds to the free end of the probe; *l *= 22 corresponds to the tethered end of the probe) have a direct bearing on the extent of the correlation coefficients between genetically unrelated probes.

#### Other types of array

It seemed unlikely that the effect was related in any way to the organism under investigation. To confirm this we analysed data from a set of ATH-121501 GeneChips (for *Arabidopsis thaliania*): as anticipated the average value of the correlation coefficient between probes with four Gs at their free ends was very high (0.86).

## Discussion

The previous section has demonstrated that probes containing a G-spot of four or more bases are very likely to be highly correlated with many other probes not in their own probe set. The phenomenon is evidently not related to genetics, so that it is clear that the pragmatic solution is simply to eliminate G-spot probes from future array designs. However, we cannot resist making some suggestions concerning the possible causes of the G-spot effect. In particular, we believe the G-spot effect results from probe-probe interactions occurring on GeneChips.

### The potential for the formation of G-quadruplexes

The high density of synthesis sites on the surface of Affymetrix GeneChips leads to crowded conditions on the array surface[9]. Assuming a stepwise synthesis yield for probes of 95% per base and that the density of initiation sites for probe synthesis is 5 × 10^17 ^molecules/m^2^, the average distance between full-length 25 mer probes is about 3 nm. As the lengths of the probes may be up to 22 nm, it is thus likely that probes can come into contact[10].

The high density of probes results in considerable differences between the rates and efficiencies of hybridisation for probes in solution and for probes tethered to a surface[11]. These differences may be due to electrostatic repulsion of the high charge density on arrays resulting from the phosphate backbones of the probes[12]. The electrostatic effects act to reduce the stability of a probe-target duplex[12] and it has been suggested[13] that probe-probe associations involving only a few residues will be able to compete with the formation of probe-target duplexes. There have been initial attempts to model probe-probe duplexes[10]. However, a full model is not computationally tractable [10] and there are presently no theoretical results which describe under what conditions probe-probe interactions occur. We believe the co-ordinated behaviour of G-spot probes results not from a probe-probe dimer but from a higher-order binding of four DNA strands.

The Hoogsteen hydrogen-bonded guanine (G)-tetrad is a four-stranded DNA spiral stack held together by eight hydrogen bonds per level[8]. Even G-quadruplexes formed by quite short runs of Gs along the 4 DNA strands can be thermally stable up to 90°C [14]. G-quadruplexes are stabilised by positive sodium or potassium cations centrally placed between adjacent (G)-tetrads. The cations are thus close to four electronegative oxygens in the (G)-tetrad above and four more in the (G)-tetrad below and act to reduce the repulsion of the oxygen atoms via the formation of cation-dipole interactions. We suggest that probes in close proximity which contain a run of four or more contiguous guanines, may sometimes interact to form a G-quadruplex.

It has been argued[7] that probes do not form G-quadruplexes on GeneChips because the probes are immobilised and so it must be the targets that form quadruplexes which cause G-spot probes to show abnormal binding. However, since the probes are sufficiently close to each other, and attached via linkers, they have enough flexibility to interact closely. Moreover, because the probes run in parallel and contain identical sequences, we believe that this provides an ideal opportunity for G-quadruplexes to form where there are runs of contiguous guanines. The coherence between all G-spot probes leads us to suggest that the problem lies with the probes and the GeneChip technology rather than the incoherently randomly segmented targets themselves.

### Brightness and chip-to-chip variability of the G-spot probes

The formation of a G-quadruplex will result in four probes having their guanines facing inwards towards the quadruplex. Thus these bases will not be available to hybridise with targets. Yet probes starting with GGGG are on average about twice as bright as other strongly correlated probes whilst containing only an average number of Cs and Gs.

We suggest the fact that G-spot probes tend to be bright may be due to the nature of the hybridisation on the surface of GeneChips resulting from the high packing density of probes. Models of the hybridisation dynamics of surface-immobilised DNA[15] show that as probes interact more strongly so the nucleation sites available are modified with resulting changes in the hybridisation affinity related to the packing density of probes. When further apart the affinity between probe and target increases rapidly. The effective association rate is proportional to (probe density)^-1.8^. We suggest that, on the surface of a chip, in a G-spot region, there will be a number of probes that form G-quadruplexes. The G-quadruplex acts to bind four probes together and these probes do not hybridise to the target. This means that the remaining probes have more space and will have increased target affinity due to a lower probe density (c.f. Figures 4 and 5 of [11]) Indeed the run of Gs on the remaining probes is available to act as an efficient nucleation site for hybridisation. This could encourage non-specific binding of labelled targets.

### Implications for the use of existing GeneChips

Our findings have several implications. The extent to which a particular 25-base sequence will form probe-probe interactions may depend upon a range of factors which vary from experiment to experiment. Thus probe-probe interactions need to be taken into account when modelling the affinity of the probe. We have detected the G-spot effect from studying the values of the correlation coefficient for pairs of probes. We have thereby identified thousands of genetically unrelated probes whose values change coherently from sample to sample. We suggest that there is one or more aspect to the preparation of each GeneChip and/or sample which may affect the extent of the formation of G-quadruplexes across the whole GeneChip. There are many things which effect the stability of quadruplexes. These include monovalent cations. Potassium has a larger affinity for a quadruplex than sodium. (However sodium is likely to be the dominant cation during hybridisation). Conversely lithium acts to destabilise G-quadruplexes. Molecular crowding also helps to induce quadruplex formation[16]. (However we suggest this should be constant from chip to chip). Ethanol has recently been shown to be a better inducer of quadruplexes than even potassium cations[17] (ethanol is used in the preparation of nucleic acids). Even the life-history of the chip, such as whether it has been stored at low/high temperatures, or preheating the Chip prior to hybridisation, may all alter the population of quadruplexes on the surface of the chip.

## Conclusion

We have shown that probes containing G-spots are typically highly correlated with each other and uncorrelated with the other members of their own probesets. When one of these probes has a high intensity it is therefore likely that other G-spot probes have high intensities. Thus a high intensity for a G-spot probe cannot be regarded as evidence that its target gene is highly expressed. Of course the G-spot probe will stand out as an outlier if the other probes in its probeset give a contradictory impression and this will cause no problem. However, if a G-spot probe has a high value (because on this array G-spot probes happen to have high values) and the other probes in the probe set have high values (because the gene is well expressed) then the G-spot probe will not be excluded in calculations of overall gene expression, even though its value is not affected by the gene, but by the conditions under which the array was treated. It is important not to rely on outlier detection procedures to throw out misleading G-spot values. The truly misleading values are those that appear to tentatively support others in their probe set. Designers of future high-density oligonucleotide arrays need to avoid runs of contiguous guanines and any other such sequences that act to stabilise probe-probe interactions between pairs of otherwise unrelated probes. For existing designs, G-spot probes should be eliminated from consideration before the analysis commences.

## Methods

During 2007 we downloaded CEL files from the NCBI Gene Expression Omnibus (GEO) repository. By the end of that year we had tens of thousands of CEL files, including 6685 examples of the most popular GeneChip produced by Affymetrix for the human genome: the HG-U133A array. These CEL files, from 162 separate GEO series (GSE) of experiments, were created between January 2002 and February 2006 and subsequently uploaded to GEO by many independent experimenters.

The next step was to create "heatmaps" (such as Fig. 1) illustrating the values of the correlation coefficients (in the log space) between all pairs of probes within each probe set. These were created using information from all the 6685 CEL files. Each CEL file was separately log normalised and potential spatial flaws identified[18]. To avoid problems with results being dominated by a few outliers we excluded data for each probe if they were more than three standard deviations from the probe's mean. Secondly we excluded not only data flagged as potentially part of a spatial flaw but also data within 60 *μ*m of a spatial flaw. Even after this ultra-cautious treatment, we had many thousands of data for each of approximately half a million probes. The resulting 22 299 visualisations are at . Inspection of the heatmaps provided an efficient method for identifying probes that, despite having reasonable average magnitudes, had low values of the correlation coefficient when paired with other probes included in their subset.

When calculating the correlation coefficient between probes containing runs of Gs, firstly only PM and MM probes with a single sequence of 2 or more Gs were selected. These were then divided into subgroups according to the length of the run of Gs and the location of the first G in the sequence. To avoid inflating the average correlation coefficient by including probes that would have been expected to be correlated in the absence of the G-spot effect, within each subgroup only the first probe in any probe set was used. Similarly where probes have identical sequences, only one was include in the averages. In Tables 1, 2 and 3 all possible correlation coefficients between pairs of probes were calculated and averaged.

## Authors' contributions

WBL was responsible for the creation of the heatmaps and drew attention to the misbehaving probes, and for the numerical results reported here. GJGU was responsible for the attribution of the misbehavior to the presence of G-spots. APH directed the research and was responsible for assembling the information on G-quadruplexes. All authors read and approved the final manuscript.
